# 免疫分型对非小细胞肺癌新辅助疗效的影响及机制

**DOI:** 10.3779/j.issn.1009-3419.2025.106.31

**Published:** 2025-11-20

**Authors:** WU Li, YANG Liying, ZHAO Miaoqing, SUN Jian, CAO Fanghan, CHEN Qianhui, SUN Xiaorong, XING Ligang

**Affiliations:** ^1^646099 泸州，西南医科大学附属医院肿瘤科（伍力，邢力刚）; ^1^Department of Oncology, The Affiliated Hospital of Southwest Medical University, Luzhou 646099, China; ^2^250100 济南，山东大学肿瘤中心（杨丽颖）; ^2^DShandong University Cancer Center, Jinan 250100, China; ^3^250117 济南，山东省肿瘤医院放射肿瘤科，山东第一医科大学及山东省医学科学院（杨丽颖，曹芳菡，陈千惠，邢力刚）; ^3^Department of Radiation Oncology, Shandong First Medical University and Shandong Academy of Medical Sciences, Jinan 250117, China; ^4^250117 济南，山东省肿瘤医院病理科，山东第一医科大学及山东省医学科学院（赵苗青）; ^4^Department of Pathology, Shandong First Medical University and Shandong Academy of Medical Sciences, Jinan 250117, China; ^5^250117 济南，山东省肿瘤医院胸外科，山东第一医科大学及山东省医学科学院（孙健）; ^5^Department of Thoracic Surgery, Shandong First Medical University and Shandong Academy of Medical Sciences, Jinan 250117, China; ^6^250117 济南，山东省肿瘤医院核医学科，山东第一医科大学及山东省医学科学院（孙晓蓉）; ^6^Department of Nuclear Medicine, Shandong First Medical University and Shandong Academy of Medical Sciences, Jinan 250117, China

**Keywords:** 肺肿瘤, 免疫浸润分型, 新辅助免疫治疗, 空间接近性, Lung neoplasms, Immune phenotypes, Neoadjuvant immunotherapy, Spatial proximity

## Abstract

**背景与目的:**

免疫治疗反应率低，可部分归因于异质性肿瘤微环境导致的肿瘤免疫逃逸机制，本研究旨在通过组织学层面确定炎症型非小细胞肺癌（non-small cell lung cancer, NSCLC）对新辅助免疫联合化疗疗效的影响，并探究特定CD8^+^ T及CD4^+^ T细胞的数量和空间接近性在疗效预测中的价值。

**方法:**

回顾性纳入2021年1月至2023年6月在山东省肿瘤医院接受新辅助免疫联合化疗的43例NSCLC患者，收集术前活检标本并进行多重免疫荧光染色[CD8/程序性细胞死亡受体1（programmed cell death protein 1, PD-1）/T细胞免疫球蛋白及黏蛋白结构域蛋白 3（T-cell immunoglobulin and mucin-domain containing protein 3, TIM-3）/CD4/叉头框蛋白3（forkhead box protein 3, FoxP3）/细胞角蛋白（cytokeratin, CK）/4',6-二脒基-2-苯基吲哚（4',6-diamidino-2-phenylindole, DAPI）]。使用InForm软件行组织分割（上皮区和间质区），并量化肿瘤细胞、CD8^+^ T细胞及分群（细胞毒性、预耗竭和耗竭）、CD4^+ ^T细胞及分群（常规和调节性）的密度及其空间接近性。基于CD8^+ ^T细胞在上皮及间质区中的相对浸润程度，将NSCLC分为炎症型（上皮区及间质区均>10/1000）、排除型（上皮区<10/1000，间质区>10/1000）和荒漠型（上皮区及间质区均<10/1000）。使用*Kolmogorov-Smirnov*检验、*Fisher's*精确检验、*Mann-Whitney U*检验及*Logistic*回归确定与主要病理缓解（major pathological response, MPR）有关的因素。

**结果:**

炎症型、排除型及荒漠型NSCLC分别占65.1%、27.9%和7.0%。与非炎症型NSCLC患者相比，炎症型MPR率更高（71.4% *vs* 33.3%, *P*=0.016）。单因素以及多因素*Logistic*回归分析均证实炎症型是NSCLC患者获得MPR的独立保护因素（OR=0.20，95%CI: 0.05-0.74，*P*=0.020；校正后OR=0.17，95%CI: 0.03-0.80，*P*=0.030）。在炎症型NSCLC上皮区内分析CD8^+ ^T与CD4^+ ^T细胞空间距离时发现，MPR组上皮区内调节性CD4^+ ^T细胞30 *μ*m半径内细胞毒性CD8^+ ^T细胞的有效密度低于non-MPR组（0.00 *vs* 0.33, *P*=0.037）。

**结论:**

炎症型NSCLC患者接受新辅助免疫联合化疗的疗效更佳，这可能与调节性CD4^+^ T细胞与细胞毒性CD8^+^ T细胞接近性减弱有关。

## Abstract (en)

**Background and objective:**

The low response rate to immunotherapy can be partially attributed to tumor immune escape mechanisms arising from the heterogeneous tumor microenvironment. This study aims to determine the impact of inflammatory non-small cell lung cancer (NSCLC) on the efficacy of neoadjuvant immunotherapy combined with chemotherapy at the histological level, and to investigate the predictive value of specific CD8^+^ and CD4^+ ^T cell numbers, as well as spatial interactions, in treatment response.

**Methods:**

A retrospective study included 43 patients with NSCLC who underwent neoadjuvant immunotherapy combined with chemotherapy at Shandong Cancer Hospital from January 2021 to June 2023. Preoperative biopsy specimens were collected and subjected to multiplex immunofluorescence staining [CD8/programmed cell death protein 1 (PD-1)/T cell immunoglobulin and mucin-domain containing protein 3 (TIM-3)/CD4/ forkhead box protein 3 (FoxP3)/cytokeratin (CK)/4',6-diamidino-2-phenylindole (DAPI)]. InForm software was used to perform tissue segmentation (epithelial and stromal regions) and quantify the density and spatial proximity of tumor cells, CD8^+ ^T cells and their subsets (cytotoxic, pre-exhausted and exhausted), as well as CD4^+ ^T cells and their subsets (conventional and regulatory). NSCLC was classified into three subtypes based on the relative infiltration levels of CD8^+^ T cells in both the epithelial and stromal compartments: inflamed (both compartments>10/1000), excluded (epithelial compartment<10/1000 and stromal compartment>10/1000), and desert (both compartments<10/1000). The *Kolmogorov-Smirnov* test, *Fisher's exact *test, *Mann-Whitney U* test and *Logistic* regression were used to identify factors associated with major pathological response (MPR).

**Results:**

Inflamed, excluded, and desert NSCLC accounted for 65.1%, 27.9% and 7.0%, respectively. Compared with patients with non-inflamed NSCLC, those with inflamed NSCLC exhibit a higher MPR rate (71.4% *vs* 33.3%, *P*=0.016). Both univariate and multivariate *Logistic *regression analyses confirmed that the inflamed subtype is an independent protective factor against the acquisition of MPR in NSCLC patients (OR=0.20, 95%CI: 0.05-0.74, *P*=0.020; adjusted OR=0.17, 95%CI: 0.03-0.80, *P*=0.030). Analysis of the spatial distance between CD8^+^ and CD4^+ ^T cells within the epithelial regions of inflamed NSCLC revealed that the effective density of cytotoxic CD8^+^ T cells within a 30 *μ*m radius of regulatory CD4^+ ^T cells was lower in the MPR group than in the non-MPR group (0.00 *vs* 0.33, *P*=0.037).

**Conclusion:**

Patients with inflamed NSCLC demonstrate superior efficacy when receiving neoadjuvant immunotherapy combined with chemotherapy. This may be due to reduced proximity between regulatory CD4^+ ^T cells and cytotoxic CD8^+^ T cells.

以免疫检查点抑制剂（immune checkpoint inhibitors, ICIs）为代表的新辅助免疫治疗方案显著改善了非小细胞肺癌（non-small cell lung cancer, NSCLC）患者的临床疗效^[1-5]^。然而，整合多项前瞻性及回顾性临床研究的meta分析^[6]^数据显示，仅有49.2%的患者对免疫治疗产生反应。这种反应差异可部分归因于异质性肿瘤微环境导致的肿瘤免疫逃逸机制^[7,8]^。基于对ICIs的不同反应，可根据CD8^+^ T细胞在肿瘤免疫微环境中的浸润情况，将NSCLC分为对ICIs治疗敏感的炎症型肿瘤和对ICIs治疗不敏感的非炎症型肿瘤（排除型和荒漠型）^[9-11]^。然而，炎症型NSCLC的免疫反应机制尚不明确，探索炎症型NSCLC的免疫反应机制可能有助于进一步提高新辅助免疫治疗的反应率。

研究^[12-14]^表明，CD8^+^ T细胞在持续抗原刺激下会逐渐从细胞毒性状态过渡到预耗竭状态，并最终发展为耗竭状态。因此，理论上，炎症型NSCLC的肿瘤微环境中可能存在更多功能活跃的CD8^+^ T细胞，但这一假设尚未在组织水平上得到证实。此外，考虑到CD8^+^ T细胞需要CD4^+^ T细胞的协助才能发挥强大的抗肿瘤作用，上皮细胞区域内功能活跃的CD8^+^ T细胞的有效范围内是否有足够的功能活跃的CD4^+^ T细胞仍需进一步研究。

本研究采用多重免疫荧光技术，对未经治疗的NSCLC患者原发灶中的CD8^+^ T和CD4^+^ T细胞及其功能亚群进行全自动定量分析，旨在从组织学层面揭示炎症型NSCLC对新辅助免疫联合化疗疗效的预测价值，并探究炎症型NSCLC获益的原因是否取决于上皮区域内CD8^+ ^T及CD4^+ ^T细胞的数量和空间接近性。

## 1 材料与方法

### 1.1 患者队列与组织采集

本研究为回顾性研究，连续纳入2021年1月至2023年6月在山东省肿瘤医院接受新辅助免疫联合化疗且符合标准的43例NSCLC患者，并收集福尔马林固定、石蜡包埋（formalin-fixed, paraffin-embedded, FFPE）的术前活检肿瘤样本。纳入标准：（1）病理分期为IIA-IIIB期的原发性NSCLC，分期标准根据美国癌症联合委员会（American Joint Committee on Cancer, AJCC）第8版肺癌肿瘤原发灶-淋巴结-转移（tumor-node-metastasis, TNM）分期；（2）切除的组织足够进行多重免疫荧光（multiplex immunofluorescence, mIF）染色；（3）具有切除组织的病理学检查报告。排除标准：（1）患有其他恶性肿瘤；（2）既往有自身免疫性疾病或其他感染性疾病病史。

本研究采用两种不同的分组方式：一种为将实现主要病理缓解（major pathological response, MPR）（定义为残留活肿瘤细胞少于10%）的患者归类为MPR组，其余患者则归类为non-MPR组^[15]^；另一种分组方式为将手术时原发肿瘤及采样淋巴结中残留活肿瘤细胞比例为0%的患者归类为病理完全缓解（pathological complete response, pCR）组，其余患者则归类为non-pCR组^[16]^。本项研究使用MPR作为最终结局进行分析。

本研究已获得山东省肿瘤医院伦理审查委员会批准（No.SDTHEC2023003055），且本研究为回顾性研究，无需患者知情同意。

### 1.2 mIF染色

对FFPE的NSCLC患者术前活检样本进行厚度为3 *μ*m的连续切片。首先，切片于60 ^o^C恒温箱过夜烤片，在二甲苯中脱蜡、梯度乙醇复水处理后，于97 °C烘箱中热处理20 min以激活抗原，3% H₂O₂溶液孵育以阻断组织内源性过氧化物酶活性，随后用40%山羊血清封闭30 min。然后，依次进行一抗、二抗孵育（opal polymer HRP Ms+Rb, ARH1001EA）和酪酰胺信号放大（tyramine signal amplification, TSA）显色，随后进行下一轮染色，与之前步骤相同。使用CD8（Ab93278, Abcam, Opal 780, 1:400）、程序性细胞死亡受体1（programmed cell death protein 1, PD-1）（ZM-0381, Zsgb-bio, Opal 620, 1:1）、T细胞免疫球蛋白及黏蛋白结构域蛋白3（T-cell immunoglobulin and mucin-domain containing protein 3, TIM-3）（45208S, Cell Signaling Technology, Opal 520, 1:200）、CD4（Ab133616, Abcam, Opal 540, 1:200）、叉头框蛋白3（forkhead box protein 3, FoxP3）（AB20034, Abcam, Opal 650, 1:100）和细胞角蛋白（cytokeratin, CK）（ZM-0069, Zsgb-bio, Opal 480, 1:100）对抗原进行标记。最后，使用4',6-二脒基-2-苯基吲哚（4',6-diamidino-2-phenylindole, DAPI）对细胞核进行染色标记。所有抗体均经过制造商验证，并于实验中使用已知阳性的淋巴结组织作为阳性对照。通过Vectra成像系统（Vectra Polaris 1.0.10）10倍物镜获取全片扫描图像。

### 1.3 多光谱成像与表型分析

对于获得的扫描图像，随机选择2个大小一致的区域作为感兴趣区域（region of interests, ROIs），以20倍放大倍率进行精准扫描，使用InForm软件（版本2.4.2）对采集的图像中的ROIs进行全自动定量分析。首先，使用InForm软件的光谱库功能，通过提取纯光谱特征，从每张图像中识别并扣除组织自发荧光。其次，依次进行组织分割、细胞分割及细胞表型识别。随后，所有图像均用该训练算法进行处理。从InForm获取的数据进一步通过R软件（版本3.6.3）进行分析。基于空间位置和表型，对每个选定区域中的所有阳性细胞进行定量分析，并通过标志物的共定位识别细胞，包括：肿瘤细胞（CK^+^）、CD8^+^ T细胞（CK^-^/CD4^-^/CD8^+^/PD-1^±^/TIM-3^±^）、细胞毒性CD8^+^ T细胞（cytotoxic CD8^+^ T cells, CD8^+^ T_cyt_）（CK^-^/CD4^-^/CD8^+^/PD-1^-^/TIM-3^-^）、预耗竭CD8^+^ T细胞（pre-dysfunctional CD8^+^ T cells, CD8^+^ T_pre）_（CK^-^/CD4^-^/CD8^+^/PD-1^+^/TIM-3^-^）、耗竭CD8^+^ T细胞（dysfunctional CD8^+^ T cells, CD8^+^ T_dys_）（CK^-^/CD4^-^/CD8^+^/PD-1^±^/TIM-3^+^）^[17]^、CD4^+^ T细胞（CK^-^/FoxP3^±^/CD4^+^/CD8^-^）、常规CD4^+^ T细胞（CD4^+^ conventional T cells, T_con_）（CK^-^/FoxP3^-^/CD4^+^/CD8^-^）和调节性CD4^+^ T细胞（CD4^+^ regulatory T cells, T_reg_）（CK^-^/FoxP3^+^/CD4^+^/CD8^-^）。各类细胞群体的数量以每1000个有核细胞中染色细胞的数量表示。采用有效密度来量化细胞间的空间接近性，其定义为以每个目标细胞为中心、30 *μ*m半径内的效应细胞的平均数量。该距离阈值的选择参考了先前关于T细胞免疫突触形成及细胞间功能性相关作用的文献，被认为足以涵盖细胞直接接触及短程信号分子扩散的有效作用范围^[18,19]^。

### 1.4 统计学方法

使用GraphPad Prism 9.0和R软件（版本4.1.2）进行统计分析。使用*Kolmogorov-Smirnov*检验确定定量数据是否符合正态分布，使用*Student’s T*检验对符合正态分布的两组数据进行组间比较，*Mann-Whitney U*检验对不符合正态分布的两个独立样本的参数进行比较。使用*Fisher's*精确检验对分类变量进行比较，*Logistic*回归评估影响治疗效果的因素。使用双侧检验，P<0.05为具有统计学差异。

## 2 结果

### 2.1 患者队列

本研究共纳入了43例接受新辅助免疫联合化疗的NSCLC患者，患者的临床病理特征见表1。以年龄>65岁（51.2%, 22/43）、男性（88.4%, 38/43）、吸烟指数>400（62.8%, 27/43）、肺鳞状细胞癌（lung squamous cell carcinoma, LUSC）（83.7%, 36/43）、IIIA期（46.5%, 20/43）、程序性细胞死亡配体1（programmed cell death-ligand 1, PD-L1）肿瘤比例评分（tumor proportion score, TPS）为1%-49%（55.8%, 24/43）的患者居多。所有患者均为表皮生长因子受体（epidermal growth factor receptor, *EGFR*）野生型。使用的PD-1抑制剂包括替雷利珠单抗（48.8%, 21/43）、信迪利单抗（20.9%, 9/43）、卡瑞利珠单抗（16.3%, 7/43）和特瑞普利单抗（14.0%, 6/43）。治疗后58.1%（25/43）患者达到了MPR，仅34.9%（15/43）患者达到pCR。此外，72.1%（31/43）患者在术后接受辅助治疗。

**表 1:** 接受新辅助免疫联合化疗的NSCLC患者的临床病理特征（*n*=43）

Characteristic	*n* (%)
Age (yr)	
≤65	21 (48.8)
>65	22 (51.2)
Gender	
Male	38 (88.4)
Female	5 (11.6)
Smoking index	
≤400	16 (37.2)
>400	27 (62.8)
cTNM stage (AJCC 8^th^)	
IIA	2 (4.7)
IIB	9 (20.9)
IIIA	20 (46.5)
IIIB	12 (27.9)
Histology	
LUSC	36 (83.7)
LUAD	7 (16.3)
PD-L1 TPS	
≤1%	1 (2.3)
1%-49%	24 (55.8)
≥50%	16 (37.2)
Not applicable	2 (4.7)
*EGFR* mutation status	
Yes	0 (0.0)
No	43 (100.0)
PD-1 inhibitors	
Tislelizumab	21 (48.8)
Sintilimab	9 (20.9)
Camrelizumab	7 (16.3)
Toripalimab	6 (14.0)
MPR	
Yes	25 (58.1)
No	18 (41.9)
pCR	
Yes	15 (34.9)
No	28 (65.1)
Postoperative adjuvant therapy	
Yes	31 (72.1)
No	12 (27.9)

NSCLC: non-small cell lung cancer; cTNM: clinical tumor-node- metastasis; AJCC: American Joint Committee on Cancer; LUSC: lung squamous cell carcinoma; LUAD: lung adenocarcinoma; EGFR: epidermal growth factor receptor; PD-1: programmed cell death protein 1; PD-L1: programmed cell death-ligand 1; TPS: tumor proportion score; MPR: major pathological response; pCR: pathological complete response.

### 2.2 肿瘤免疫浸润分型以及与临床病理特征的关系

为探究不同肿瘤免疫浸润分型的NSCLC的特征，首先通过InForm监督图像分析系统对mIF染色后图像进行组织分割、细胞分割以及T细胞识别；随后分别将上皮区和间质区内T细胞计数除以总细胞计数（细胞计数/1000细胞），以对其浸润密度进行标准化计算（图1A）。在纳入的43例患者中于上皮区和间质区分别识别出3354和23,603个T细胞。其中，CD8⁺ T细胞分别为1307个（上皮区）和4961个（间质区），CD4⁺ T细胞分别为2047个（上皮区）和18,642个（间质区）。进一步基于标志物共定位分析，识别出3个CD8⁺ T细胞亚群：CD8⁺ T_cyt_、CD8⁺ T_dys_和CD8⁺ T_pre_，这3个亚群在上皮区和间质区的细胞数分别为706与1482个、135与423个、466与3056个；同时，识别出2个CD4⁺ T细胞亚群：CD4⁺ T_con_和CD4⁺ T_reg_，在上皮区和间质区的细胞数分别为1971与16,681个、76与1961个。基于上皮区及间质区每1000个细胞中CD8^+^ T细胞的相对数量，将NSCLC患者分为炎症型、排除型和荒漠型，分别为28例（65.1%）、12例（27.9%）和3例（7.0%）。炎症型定义为上皮区和间质区CD8^+^ T细胞均>10/1000；排除型定义为上皮区CD8^+^ T细胞<10/1000，而间质区CD8^+^ T细胞>10/1000；荒漠型定义为上皮区和间质区CD8^+^ T细胞均<10/1000^[9,20]^。图1B为3种肿瘤免疫浸润分型的代表性图像。此外，因荒漠型病例数较少（*n*=3），为避免统计偏倚，将其与排除型合并为非炎症型进行后续分析，该处理可能掩盖两种亚型间的生物学差异。对于纳入的43例NSCLC患者，炎症型与非炎症型NSCLC患者的性别、年龄、临床TNM（clinical TNM, cTNM）分期、病理类型和PD-L1 TPS这些临床病理特征无显著差异（表2）。

**图 1:**
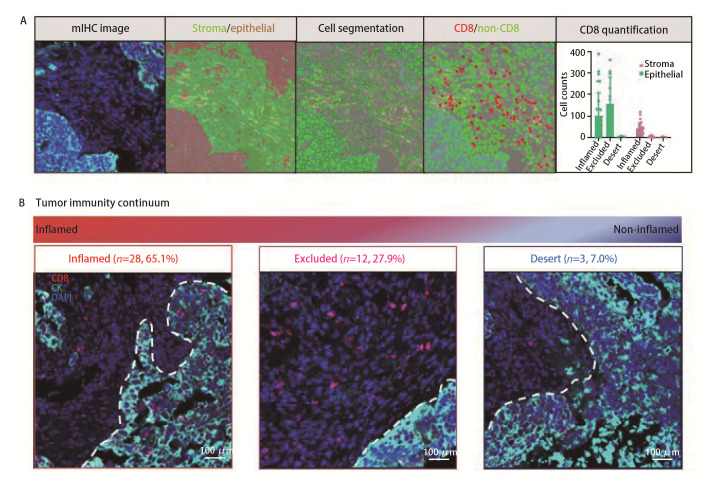
构建肿瘤免疫浸润分型。A：采用mIF技术在NSCLC组织中识别CD8^+^ T细胞；B: 根据上皮和间质区中CD8^+^ T细胞含量不同，将肿瘤分为炎症型、排除型和荒漠型。

**表 2:** 肿瘤免疫浸润分型与临床病理特征的关系

Characteristic	*n*	Inflamed (*n*=28)	Non-inflamed (*n*=15)	P*
Age (yr)				0.203
≤65	21	16 (57.1%)	5 (33.3%)	
>65	22	12 (42.9%)	10 (66.7%)	
Gender				0.145
Male	38	23 (82.1%)	15 (100.0%)	
Female	5	5 (17.9%)	0 (0.0%)	
cTNM stage (AJCC 8^th^)				0.196
IIA	2	2 (7.1%)	0 (0.0%)	
IIB	9	6 (21.4%)	3 (20.0%)	
IIIA	20	10 (35.7%)	10 (66.7%)	
IIIB	12	10 (35.7%)	2 (13.3%)	
Histology				0.680
LUSC	36	24 (85.7%)	12 (80.0%)	
LUAD	7	4 (14.3%)	3 (20.0%)	
PD-L1 TPS				0.835
≤1%	1	1 (3.6%)	0 (0.0%)	
1%-49%	24	14 (50.0%)	10 (66.7%)	
≥50%	16	11 (39.2%)	5 (33.3%)	
Not applicable	2	2 (7.2%)	0 (0.0%)	

*The P-values were calculated using Fisher’s exact test.

### 2.3 炎症型NSCLC与治疗反应的关系

对不同免疫浸润分型NSCLC患者治疗反应的分析发现，炎症型NSCLC患者的MPR率高于非炎症型（71.4% vs 33.3%, *P*=0.016）。单因素及多因素逻辑回归分析（表3）显示，炎症型是NSCLC患者获得MPR的独立保护因素（OR=0.20，95%CI: 0.05-0.74，*P*=0.020；校正后OR=0.17，95%CI: 0.03-0.80，*P*=0.030）。

**表 3:** 相关因素与治疗反应的Logistic回归分析

Item	Univariate analysis				Multivariate analysis		
	OR	95%CI	*P*		OR	95%CI	*P*
Age (≤65 vs >65 yr)	0.50	0.14-1.69	0.271		0.53	0.11-2.45	0.422
Gender (Male vs Female)	0.43	0.05-2.92	0.391		0.28	0.02-4.17	0.346
Smoking index (≤400 vs >400)	0.75	0.20-2.63	0.656		0.97	0.14-6.24	0.972
cTNM stage (III vs II)	2.35	0.56-12.26	0.263		2.19	0.38-15.36	0.393
Histology (LUAD vs LUSC)	0.23	0.03-1.21	0.101		0.32	0.03-2.43	0.290
IP (Inflamed vs Non-inflamed)	0.20	0.05-0.74	0.020		0.17	0.03-0.80	0.030

IP: immune phenotype; OR: odds ratio; CI: confidence interval.

### 2.4 炎症型NSCLC肿瘤区域内的细胞组成与治疗反应的关系

为探索炎症型NSCLC在新辅助免疫联合化疗后治疗反应更佳的原因，进一步对炎症型NSCLC患者上皮区内的细胞组成进行分析。图2A为mIF染色后标志物的代表性图像，根据标志物的共表达情况鉴定不同功能状态的T细胞。在炎症型NSCLC患者的上皮区，CD8^+ ^T和CD4^+^ T细胞分别占所有细胞的11.65%和19.40%。此外，还鉴定出3个CD8^+ ^T细胞分群[CD8^+^ T_cyt _(49.36%), CD8^+^ T_dys _(45.30%), CD8^+^ T_pre _(5.34%)]和2个CD4^+ ^T细胞功能分群[CD4^+^ T_con _(98.38%), CD4^+^ T_reg _(1.62%)]，见图2B。在炎症型NSCLC患者上皮区内，MPR与non-MPR组患者总CD8^+^ T细胞占比在两组间差异接近统计学意义（*P*=0.063，表4），提示可能存在趋势，但当前样本量可能不足以检测出显著差异，而CD4^+^ T细胞的数量无差异（表4，图2C）。进一步分析CD8^+ ^T细胞分群（细胞毒性、预耗竭和耗竭）和CD4^+ ^T细胞分群（常规和调节性）的构成亦无统计学差异（图2D，表4）。

**图 2:**
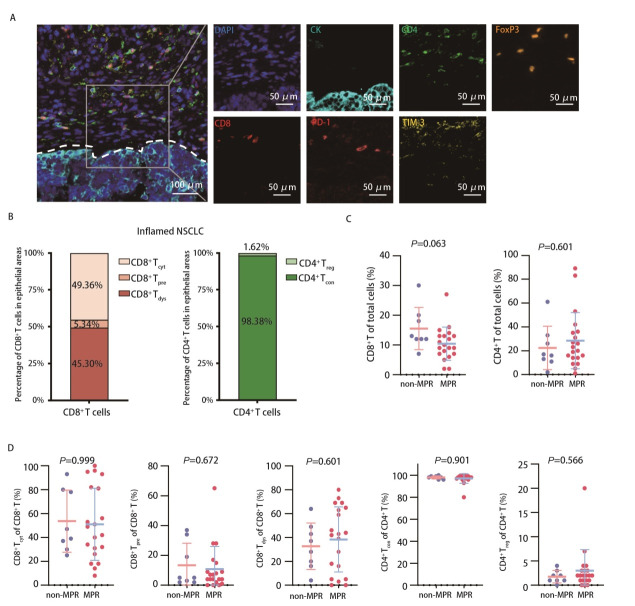
评估炎症型NSCLC上皮区内T细胞组成特征。A：mIF：DAPI/CK/CD4/FoxP3/CD8/PD-1/TIM-3的多光谱融合图像；B：炎症型NSCLC上皮区内CD8^+^ T细胞与CD4^+^ T细胞的比例；C：炎症型NSCLC上皮区内CD8^+^ T与CD4^+^ T细胞与治疗反应的关系；D：炎症型NSCLC上皮区内CD8^+ ^T和CD4^+^ T细胞的功能亚群与治疗反应的关系。

**表 4:** 炎症型NSCLC中non-MPR组和MPR组的上皮区域细胞组成的差异

Effective density	non-MPR	MPR	P*
CD8^+ ^T/total cell (epi)	13 (12, 19)	10 (7, 13)	0.063
CD4^+ ^T/total cell (epi)	16 (12, 28)	22 (14, 35)	0.601
CD8^+ ^T_cyt_/CD8^+ ^T (epi)	43 (35, 79)	46 (30, 81)	0.999
CD8^+ ^T_pre_/CD8^+ ^T (epi)	5 (3, 23)	6 (4, 11)	0.672
CD8^+ ^T_dys_/CD8^+ ^T (epi)	33 (19, 45)	43 (13, 64)	0.601
CD4^+ ^T_con_/CD4^+ ^T (epi)	98 (98, 99)	98 (97, 99)	0.901
CD4^+ ^T_reg_/CD4^+ ^T (epi)	2 (1, 2)	2 (1, 3)	0.566

epi: epithelial; CD8^+^ T_cyt_: cytotoxic CD8^+^ T; CD8^+^ T_pre_: pre-dysfunctional CD8^+^ T; CD8^+^ T_dys_: dysfunctional CD8^+^ T; CD4^+ ^T_con_: CD4^+ ^conventional T; CD4^+^ T_reg_: CD4^+ ^regulatory T. All data are expressed as median (Q1, Q3). *The P-values were calculated using the Mann-Whitney U test.

### 2.5 炎症型NSCLC肿瘤区域内细胞间的空间接近性与治疗反应的关系

CD8^+^ T细胞可能需要与CD4^+^ T细胞合作以引发强烈的抗肿瘤反应^[21,22]^，然而，目前尚不清楚在具有更高治疗反应率的炎症性NSCLC的上皮区内CD8^+^ T细胞与CD4^+^ T细胞的空间接近性是否增加，因此本研究中使用有效密度来评估CD8^+^ T细胞与CD4^+^ T细胞的空间接近性。有效密度定义为以每个CD4^+^ T细胞为中心的一定半径范围内存在的CD8^+^ T细胞的平均数量（图3A）。有效密度高表明空间接近性程度高，而有效密度低则提示空间接近性较弱。

**图 3:**
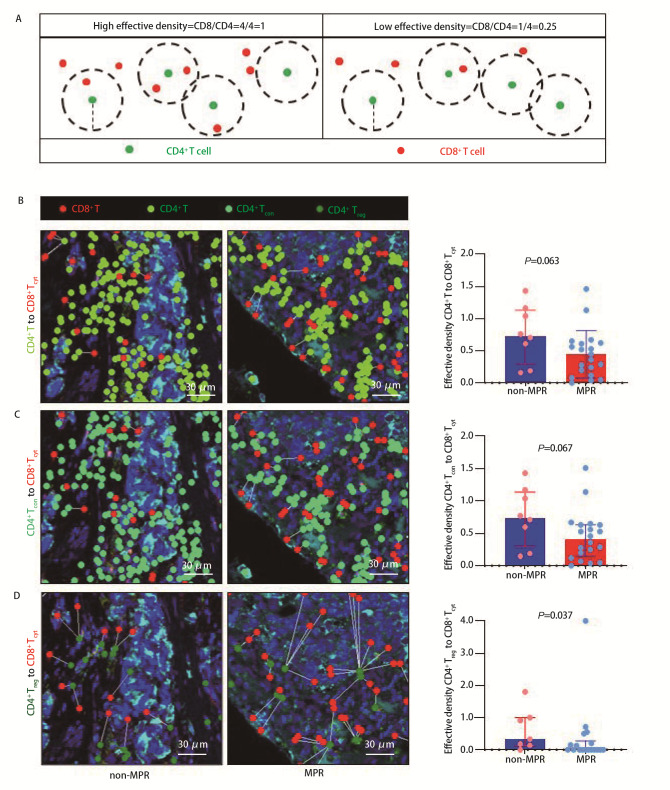
CD4^+^ T与CD8^+^ T细胞之间的空间接近性及其对NSCLC患者新辅助免疫联合化疗治疗反应的影响。A：高和低有效密度的示意图；B：炎症型NSCLC上皮区内CD4^+^ T细胞-CD8^+ ^T_cyt_细胞的有效密度对治疗反应的影响；C：炎症型NSCLC上皮区内CD4^+^ T_con_-CD8^+^ T_cyt_有效密度对治疗反应的影响；D：炎症型NSCLC上皮区内CD4^+^ T_reg_-CD8^+^ T_cyt_有效密度对治疗反应的影响。左：代表性图像；右：散点图。P值采用Mann-Whitney U检验计算。

在炎症型NSCLC的上皮区内，MPR组与non-MPR组患者不同范围内的CD4^+^ T细胞和CD8^+^ T细胞的有效密度无统计学差异。进一步对细胞亚群的分析显示，MPR组与non-MPR组在CD4^+^ T细胞、CD4^+^ T_con_与CD8^+^ T_cyt_的有效密度上无显著差异（图3B、3C）。然而，MPR组上皮区内CD4^+^ T_reg_周围30 *μ*m半径内CD8^+^ T_cyt_的有效密度低于non-MPR组（0.00 vs 0.33，*P*=0.037，图3D，表5）。此外，MPR组与non-MPR组之间CD4^+^ T_con_和CD4^+^ T_reg_与CD8^+^ T细胞、CD8^+^ T_pre_及CD8^+^ T_dys_的有效密度无统计学差异（表5）。

**表 5:** 炎症型NSCLC中non-MPR组与MPR组有效密度的差异

Effective density (epithelial)	non-MPR	MPR	P*
CD4^+^ T to CD8^+^ T_10 *μ*m	0.22 (0.14, 0.38)	0.14 (0.03, 0.20)	0.063
CD4^+^ T_con _to CD8^+^ T_10 *μ*m	0.22 (0.13, 0.38)	0.14 (0.03, 0.21)	0.063
CD4^+^ T_reg_ to CD8^+ ^T_10 *μ*m	0.29 (0.10, 0.72)	0.02 (0.00, 0.29)	0.229
CD4^+^ T to CD8^+^ T_cyt__10 *μ*m	0.07 (0.04, 0.13)	0.04 (0.01, 0.07)	0.066
CD4^+ ^T_con_ to CD8^+ ^T_cyt__10 *μ*m	0.08 (0.04, 0.13)	0.04 (0.01, 0.07)	0.059
CD4^+ ^T_reg_ to CD8^+ ^T_cyt__10 *μ*m	0.00 (0.00, 0.00)	0.00 (0.00, 0.00)	0.924
CD4^+^ T to CD8^+^ T_pre__10 *μ*m	0.00 (0.00, 0.05)	0.00 (0.00, 0.01)	0.672
CD4^+ ^T_con_ to CD8^+ ^T_pre__10 *μ*m	0.00 (0.00, 0.05)	0.00 (0.00, 0.01)	0.405
CD4^+ ^T_reg_ to CD8^+ ^T_pre__10 *μ*m	0.00 (0.00, 0.00)	0.00 (0.00, 0.00)	0.368
CD4^+ ^T to CD8^+ ^T_dys__10 *μ*m	0.14 (0.08, 0.25)	0.07 (0.00, 0.13)	0.170
CD4^+ ^T_con_ to CD8^+ ^T_dys__10 *μ*m	0.14 (0.08, 0.25)	0.07 (0.00, 0.13)	0.162
CD4^+ ^T_reg_ to CD8^+ ^T_dys__10 *μ*m	0.20 (0.09, 0.72)	0.00 (0.00, 0.21)	0.110
CD4^+^ T to CD8^+^ T_20 *μ*m	0.70 (0.46, 1.33)	0.50 (0.26, 0.74)	0.088
CD4^+^ T_con _to CD8^+^ T_20 *μ*m	0.70 (0.46, 1.33)	0.49 (0.27, 0.75)	0.088
CD4^+^ T_reg_ to CD8^+ ^T_20 *μ*m	0.86 (0.49, 2.61)	0.42 (0.00, 0.97)	0.145
CD4^+^ T to CD8^+^ T_cyt__20 *μ*m	0.36 (0.22, 0.44)	0.18 (0.09, 0.25)	0.053
CD4^+ ^T_con_ to CD8^+ ^T_cyt__20 *μ*m	0.36 (0.22, 0.45)	0.19 (0.09, 0.26)	0.063
CD4^+ ^T_reg_ to CD8^+ ^T_cyt__20 *μ*m	0.14 (0.06, 0.59)	0.00 (0.00, 0.00)	0.035
CD4^+^ T to CD8^+^ T_pre__20 *μ*m	0.03 (0.01, 0.20)	0.01 (0.00, 0.07)	0.170
CD4^+ ^T_con_ to CD8^+ ^T_pre__20 *μ*m	0.03 (0.01, 0.20)	0.01 (0.00, 0.07)	0.155
CD4^+ ^T_reg_ to CD8^+ ^T_pre__20 *μ*m	0.00 (0.00, 0.00)	0.00 (0.00, 0.04)	0.339
CD4^+ ^T to CD8^+ ^T_dys__20 *μ*m	0.43 (0.16, 0.69)	0.28 (0.01, 0.47)	0.413
CD4^+ ^T_con_ to CD8^+ ^T_dys__20 *μ*m	0.43 (0.16, 0.69)	0.28 (0.01, 0.47)	0.399
CD4^+ ^T_reg_ to CD8^+ ^T_dys__20 *μ*m	0.53 (0.36, 1.85)	0.09 (0.00, 0.73)	0.125
CD4^+^ T to CD8^+^ T_30 *μ*m	1.47 (1.00, 2.59)	0.99 (0.50, 1.54)	0.071
CD4^+^ T_con _to CD8^+^ T_30 *μ*m	1.47 (1.00, 2.59)	0.99 (0.51, 1.53)	0.071
CD4^+^ T_reg_ to CD8^+ ^T_30 *μ*m	1.14 (0.94, 4.60)	0.87 (0.05, 1.31)	0.190
CD4^+^ T to CD8^+^ T_cyt__30 *μ*m	0.73 (0.50, 1.07)	0.41 (0.18, 0.61)	0.063
CD4^+ ^T_con_ to CD8^+ ^T_cyt__30 *μ*m	0.74 (0.50, 1.07)	0.41 (0.18, 0.63)	0.067
CD4^+ ^T_reg_ to CD8^+ ^T_cyt__30 *μ*m	0.33 (0.16, 0.96)	0.00 (0.00, 0.19)	0.037
CD4^+^ T to CD8^+^ T_pre__30 *μ*m	0.05 (0.03, 0.38)	0.04 (0.01, 0.15)	0.210
CD4^+ ^T_con_ to CD8^+ ^T_pre__30 *μ*m	0.06 (0.03, 0.39)	0.04 (0.01, 0.16)	0.210
CD4^+ ^T_reg_ to CD8^+ ^T_pre__30 *μ*m	0.00 (0.00, 0.04)	0.00 (0.00, 0.08)	0.913
CD4^+ ^T to CD8^+ ^T_dys__30 *μ*m	0.80 (0.28, 1.31)	0.54 (0.03, 0.86)	0.241
CD4^+ ^T_con_ to CD8^+ ^T_dys__30 *μ*m	0.80 (0.28, 1.30)	0.54 (0.03, 0.87)	0.241
CD4^+ ^T_reg_ to CD8^+ ^T_dys__30 *μ*m	0.71 (0.36, 3.50)	0.35 (0.00, 1.08)	0.239

All data are expressed as median (Q1, Q3). *The *P*-values were calculated using the Mann-Whitney U test.

## 3 讨论

本研究分析了T细胞的空间分布，以揭示新辅助免疫联合化疗中炎症型NSCLC的潜在反应机制。关键发现包括：炎症型NSCLC患者接受新辅助免疫联合化疗的疗效更佳，这可能与CD4^+^ T_reg_与CD8^+^ T_cyt_接近性减弱有关。尽管ICIs疗法已彻底改变了癌症治疗，但仅有半数患者能获得良好的治疗反应^[3,23]^。在实体瘤患者中，应答者表现出以T淋巴细胞浸润为特征的“热”表型，而非应答者可能表现出以肿瘤实质中T细胞缺乏为特征的“冷”表型^[7,24]^。由于ICIs疗法被发现能激活肿瘤中的CD8^+^ T细胞，因此基于CD8^+^ T淋巴细胞功能评估ICIs疗法的疗效似乎是合理的。本项研究中采用mIF技术对治疗前肿瘤的上皮区和间质区进行了全自动定量分析，并根据CD8^+^ T细胞浸润差异将肿瘤分为炎症型、排斥型和荒漠型^[8,25]^。炎症型NSCLC被发现具有更高的免疫治疗反应率，这与既往研究^[26]^一致。

研究^[9,25]^表明，尽管“热”肿瘤通常与大量T细胞浸润有关，但并非所有浸润的T细胞均对肿瘤有反应性。既往研究^[27]^表明，T_reg_通过代谢竞争直接削弱效应T细胞功能，并通过产生白细胞介素-10（interleukin-10, IL-10）和转化生长因子-β（transforming growth factor-β, TGF-β）、IL-35等抑制性细胞因子直接抑制效应T细胞的功能和增殖，同时还能促进髓源性抑制细胞的聚集和功能^[28]^。上述研究表明T_reg_参与构成了局部抑制的免疫微环境，处于这一微环境的细胞毒性CD8^+ ^T细胞抗肿瘤能力受损。本研究的数据表明，约40%的炎症型NSCLC患者对治疗仍无反应。对炎症型NSCLC中MPR与non-MPR组微环境差异的进一步分析显示，上皮区中功能不同的CD8^+^ T细胞与CD4^+^ T细胞的相对比例无显著差异。然而，CD8^+ ^T细胞与CD4^+^ T细胞之间的有效空间接近性存在差异，具体而言，抑制性CD4^+^ T_reg_细胞在应答组中与细胞毒性CD8^+^ T_cyt_的接近性较少，这表明浸润性NSCLC患者初始治疗耐药的主要原因可能是CD4^+^ T_reg_抑制了CD8^+^ T_cyt_的细胞毒性，与既往的研究结果相同。此外，本研究结果还表明即使同为炎症型NSCLC且微环境内T细胞比例相当，空间微环境的细微差异仍然可显著影响疗效，如CD4^+^ T_reg_与CD8^+^ T_cyt_的空间接近性，这肯定了空间分析在免疫微环境研究中的重要性。值得注意的是，本研究仅观察到了CD4^+^ T_reg_与CD8^+^ T_cyt_空间接近性与疗效的关联性，并未直接检测异质性分子（如IL-10、TGF-β）或检查点分子[如细胞毒性T淋巴细胞相关蛋白4（cytotoxic T lymphocyte-associated antigen-4, CTLA-4）]的表达。因此，这种空间关系是否直接导致功能抑制，尚需未来进一步验证。

本研究存在一些局限性。首先，本研究主要终点为MPR，缺乏总生存期（overall survival, OS）和无病生存期（disease-free survival, DFS）等长期预后指标，因此无法验证免疫分型和患者长期生存获益的关联，削弱了结论的远期临床价值；其次，本研究中非炎症型NSCLC患者（15例）的样本量有限，未单独对荒漠型（3例）和排除型（12例）进行分析，并且对非炎症型的分析的说服力不足。在后续的研究中，课题组将扩充样本量以验证本研究的结果，并进一步分析荒漠型和排除型的NSCLC患者治疗获益的潜在因素。此外，本研究仅使用术前活检样本进行评估，其可能无法完全代表肿瘤整体的免疫浸润状态，肿瘤内部异质性可能对免疫分型结果产生影响。最后，本研究纳入的患者使用了多种PD-1抑制剂，并且在逻辑分析中未将免疫药物种类纳入多因素模型分析，但免疫药物的种类可能影响不同类型NSCLC患者的疗效，从而对结果产生干扰。此外，本研究对非炎症型（排除型与荒漠型）的关注不足，未能解析其各自独特的免疫微环境特征，也未探讨如何通过联合策略（如加入抗血管生成药物）改善其免疫状态，这限制了本研究对广泛NSCLC人群的临床指导价值。本研究采用的有效密度指标解释了细胞空间接近性，未来研究可结合其他空间计量学指标（如细胞间最小距离）进行综合验证。总的来说，本研究为深入理解NSCLC患者在新辅助免疫联合化疗中的免疫反应提供了宝贵见解，但未来仍需扩大样本量以验证结果并探索更多潜在影响因素。

综上所述，本研究不仅揭示了炎症型NSCLC新辅助免疫联合化疗的可能反应机制，还为优化治疗策略提供了新视角。未来进行的深入研究将有助于明确具体靶点并为个性化治疗奠定基础。
